# Fatal Infection in Ferrets after Ocular Inoculation with Highly Pathogenic Avian Influenza A(H5N1) Virus

**DOI:** 10.3201/eid3007.240520

**Published:** 2024-07

**Authors:** Jessica A. Belser, Xiangjie Sun, Joanna A. Pulit-Penaloza, Taronna R. Maines

**Affiliations:** Centers for Disease Control and Prevention, Atlanta, Georgia, USA

**Keywords:** influenza, avian influenza A(H5N1), ferret, ocular, pathogenesis, viruses, transmission, highly pathogenic avian influenza, United States

## Abstract

Ocular inoculation of a clade 2.3.4.4b highly pathogenic avian influenza A(H5N1) virus caused severe and fatal infection in ferrets. Virus was transmitted to ferrets in direct contact. The results highlight the potential capacity of these viruses to cause human disease after either respiratory or ocular exposure.

In recent years, clade 2.3.4.4b highly pathogenic avian influenza A(H5N1) viruses have exhibited substantial host expansion, geographic spread, and reassortment with other circulating influenza A viruses (IAVs) in birds, resulting in epornitics on all continents and virus detection in an expanding group of mammals (*1*). Human cases of H5N1 clade 2.3.4.4b virus infection have been reported, typically following direct exposure to infected animals, contaminated environments, or both (*2*). A 2.3.4.4b highly pathogenic avian influenza A(H5N1) virus was isolated from a human patient in Chile during 2023 (A/Chile/25945/2023 [Chile/25945]) (*3*) and caused severe and fatal disease in ferrets intranasally inoculated with 10^6^ PFU of virus. Transmission of virus to animals housed in close contact was also reported (*3*), highlighting the pandemic potential of clade 2.3.4.4b viruses. 

Although the eyes represent a secondary mucosal surface that is susceptible to respiratory virus exposure (*4*), as evidenced by recent reports of conjunctivitis in 2 dairy workers exposed to clade 2.3.4.4b H5N1 virus (*5*), risk assessment approaches for clade 2.3.4.4b H5N1 viruses to date have been limited to standard intranasal inoculation (*3*,*6*) and have not evaluated the capacity of those viruses to cause disease after alternative portals of entry. To investigate relative similarities between ocular and respiratory exposure to H5N1 virus, we assessed the severity and kinetics of disease after ocular exposure of ferrets to Chile/25945 virus and compared our findings with a previously published assessment of animals intranasally inoculated with this virus at the Centers for Disease Control and Prevention in 2023 (*3*).

To assess disease severity and transmissibility under different exposure concentrations, we inoculated ferrets by the ocular route with either a high (10^6^ PFU) or low (10^3^ PFU) dose of Chile/25945 virus (*7*). At either challenge dose, all ferrets inoculated by the ocular route became productively infected, reaching mean maximum weight loss of 12.7% (high dose) and 13.2% (low dose) and mean maximum rises in temperature of 2.4°C (high dose) and 2.0°C (low dose). Humane endpoints were reached on postinoculation days 5–7 in 3/3 (high dose) and 2/3 (low dose) animals (Figure 1, panel A). During necropsy, we detected infectious virus throughout the respiratory tract and in several extrapulmonary tissues (including from the gastrointestinal tract, central nervous system, and ocular system) (Figure 1, panel C), consistent with the highly virulent phenotype observed after high-dose intranasal inoculation of ferrets (*3*). One ferret survived the challenge with a serologic titer of 160.

**Figure 1:**
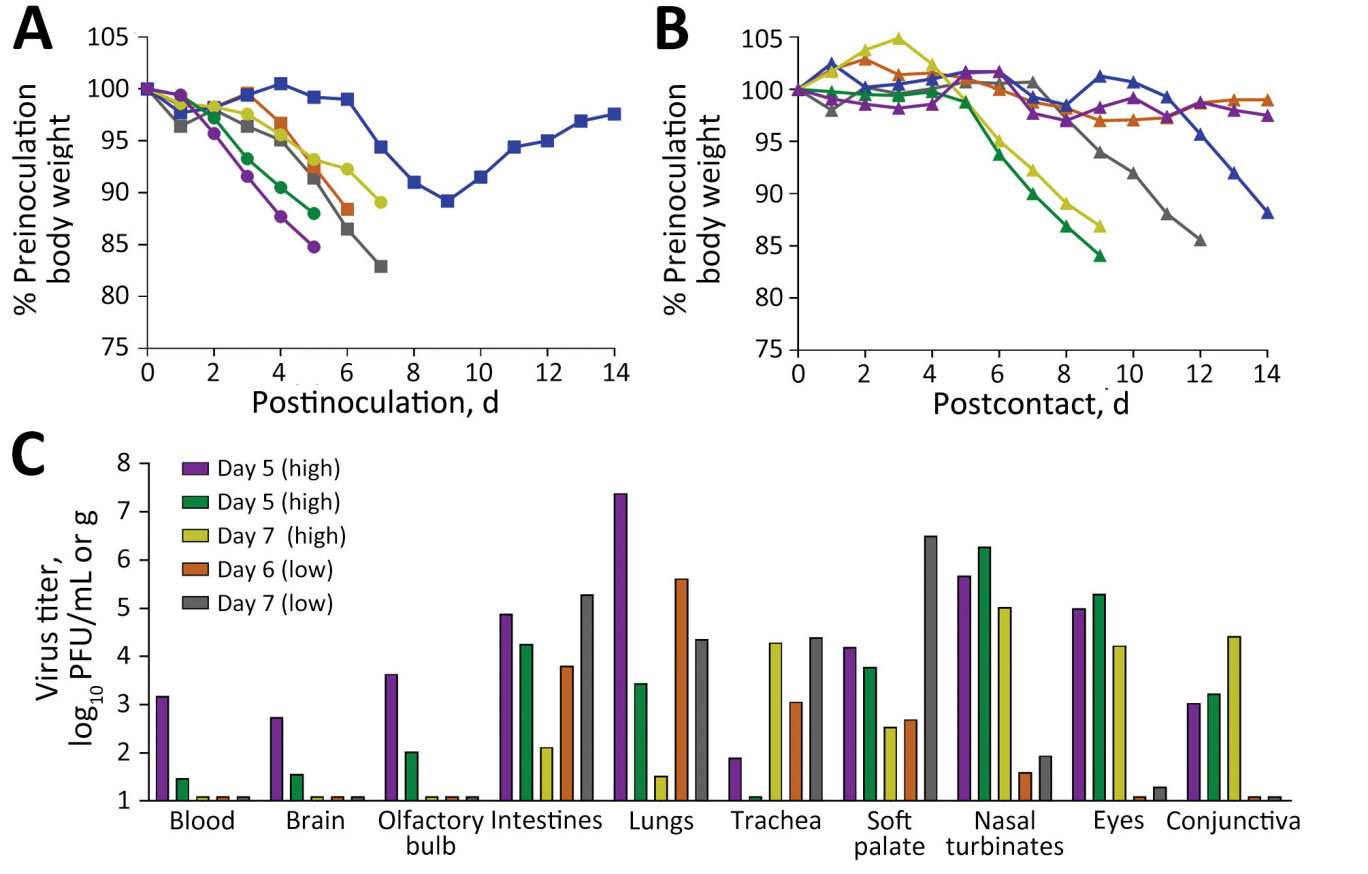
Disease severity and systemic spread of Chile/25945 influenza virus after ocular inoculation of ferrets. Ferrets were inoculated by the ocular route as previously described (*7*) with a high (10^6^ PFU, circles) or low (10^3^ PFU, squares) dose of Chile/25945 virus (100 μL volume), and each was cohoused with a serologically naive ferret 24 hours after inoculation (triangles). A, B) Inoculated (A) and contact (B) animals were weighed daily and humanely euthanized after reaching previously described endpoints (*3*). Ferret inoculated:contact pairs are indicated with shared colors. C) Systemic tissues were collected from inoculated animals that reached humane endpoints and titered for the presence of infectious virus as previously described (*7*). Bars represent individual ferrets with the postinoculation day on which humane endpoints were reached and tissues were collected specified per inoculation dose (bar color is linked with ferret morbidity data shown in panel A). Limit of detection was 10 PFU.

To assess if ferrets inoculated by the ocular route were as capable as intranasally inoculated ferrets to transmit Chile/25945 virus in a direct contact setting (*3*), we cohoused a serologically naive ferret with each inoculated ferret 24 hours after inoculation. To assess virus replication within and beyond the respiratory tract, we collected nasal wash, conjunctival wash/swab, and rectal swab samples from inoculated and contact animals. All ferrets inoculated by the ocular route with a high dose of virus had detectable infectious virus in nasal wash (mean peak titer 5.3 ± 0.2 log_10_ PFU/mL), conjunctival wash/swab (4.4 ± 0.9 log_10_ PFU/mL), and rectal swab (3.6 ± 0.3 log_10_ PFU/mL) samples (Figure 2, panel A). The magnitude and frequency of viral titers in these specimens was reduced, but still present, in animals inoculated with a low dose of virus (Figure 2, panel B). Infectious virus was detected in either nasal or rectal wash samples in all contact animals on at least 1 day; infectious virus was not detected in conjunctival wash samples from any contact animal, possibly resulting from less overall virus shedding than in inoculated animals. Humane euthanasia because of severe disease was warranted for 4/6 contact animals (Figure 1, panel B); the other 2 survived, 1 of which exhibited a low level of seroconversion (hemagglutination inhibition titer 20).

**Figure 2:**
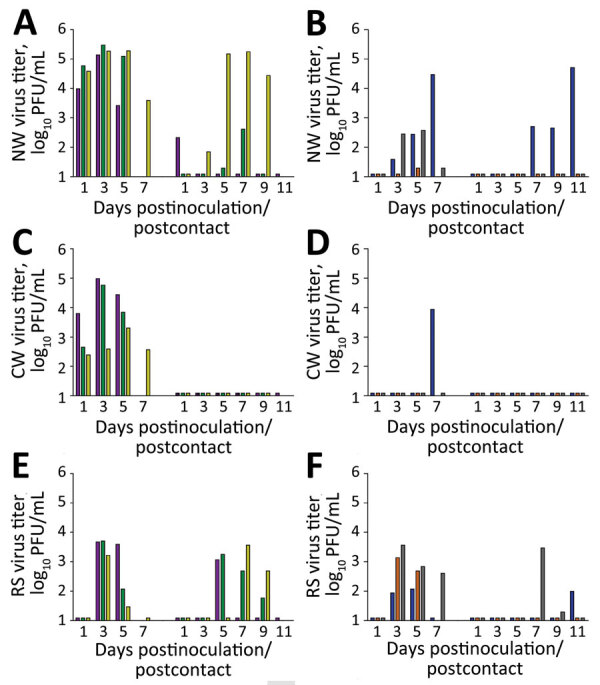
Transmission of Chile/25945 virus after ocular inoculation of ferrets. Ferrets were inoculated by the ocular route as previously described (*7*) with a high (10^6^ PFU) or low (10^3^ PFU) dose of Chile/25945 virus (100 μL), and each was cohoused with a serologically naive ferret 24 hours after inoculation. Specimens were collected from all ferrets as previously described (*7*) on alternate days after contact. A) NW specimen after ferret inoculation with 10^6^ PFU challenge dose; B) NW specimen after inoculation with 10^3^ PFU challenge dose; C) CW specimen after ferret inoculation with 10^6^ PFU challenge dose; D) CW specimen after ferret inoculation with 10^3^ PFU challenge dose; E) RS specimen after ferret inoculation with 10^6^ PFU challenge dose; F) RS specimen after ferret inoculation with 10^3^ PFU challenge dose. On each graph, left-hand bars indicate inoculated ferrets and right-hand bars indicate contact ferrets. Absence of a bar indicates an animal was humanely euthanized and no specimen was collected. Bar colors are linked with ferret morbidity data shown in Figure 1, panels A, B. Limit of detection was 10 PFU. CW, conjunctival wash/swab sample; NW, nasal wash sample; RS, rectal swab sample.

Our finding that a clade 2.3.4.4b H5N1 virus isolated from a human can exhibit a virulent and transmissible phenotype after nontraditional inoculation, even with a low dose of inoculum, underscores the public health threat posed by those IAVs. The ocular surfaces may be exposed to infectious virus from the environment by several means (e.g., airborne particles, physical transfer from contact with fomites, and splashing liquids). Furthermore, circulation of tear fluid between ocular and nasopharyngeal tissues via the lacrimal duct offers an opportunity for infectious virus to spread from the respiratory tract to the ocular system (*8*), in agreement with successful H5N1 viral isolation from both conjunctival and nasopharyngeal swab specimens from a human with conjunctivitis (*5*). Conjunctivae may be exposed to virus by direct contact (e.g., virus-contaminated hands), indirect contact (e.g., virus-contaminated fomites), or after deposition of virus-laden droplets or aerosols onto the ocular surface (*4*), permitting opportunities for H5N1 virus to establish a productive infection in humans even in the absence of an ocular tropism. Considering the myriad ways humans may be exposed to IAVs, our study supports the need to consider nontraditional inoculation modalities in risk assessment activities and supports consideration of using eye protection in potentially contaminated environments (*9*).
